# Social epidemiology of online dating in U.S. early adolescents

**DOI:** 10.1186/s13104-024-06777-w

**Published:** 2024-05-22

**Authors:** Jason M. Nagata, Priyadharshini Balasubramanian, Joan E. Shim, Jonanne Talebloo, Felicia Yen, Abubakr A.A. Al-shoaibi, Iris Yuefan Shao, Kyle T. Ganson, Alexander Testa, Orsolya Kiss, Fiona C. Baker

**Affiliations:** 1 Department of Pediatrics, University of California, San Francisco, 550 16th Street, 4th Floor, 94143 San Francisco, CA Box 0503, USA; 2 Factor-Inwentash Faculty of Social Work, University of Toronto, 246 Bloor Street W, M5S 1V4 Toronto, ON Canada; 3 Department of Management, Policy and Community Health, University of Texas Health Science Center at Houston, 7000 Fannin Street, 77030 Houston, TX USA; 4 Center for Health Sciences, SRI International, 333 Ravenswood Ave, 94025 Menlo Park, CA USA; 5 School of Physiology, University of the Witwatersrand, 1 Jan Smuts Ave, Braamfontein, 2000 Johannesburg, South Africa

**Keywords:** Adolescent, Online dating, Relationships, Dating, Social epidemiology, LGBTQ+

## Abstract

**Objective:**

To investigate the prevalence and sociodemographic associations of online dating in a demographically diverse U.S. national cohort of early adolescents.

**Methods:**

We analyzed cross-sectional data from the Adolescent Brain Cognitive Development Study (Year 2, 2018–2020, ages 11–12; *N* = 10,157). Multivariable logistic regression analyses were employed to estimate associations between sociodemographic factors (e.g., age, sex, race/ethnicity, sexual orientation, household income, parental education) and early adolescent-reported online dating behaviors.

**Results:**

Overall, 0.4% (*n* = 38) of participants reported ever using a dating app. Males (AOR 2.72, 95% CI 1.11–6.78) had higher odds of online dating compared to females, and sexual minority identification (e.g., lesbian, gay, or bisexual; AOR 12.97, 95% CI 4.32–38.96) was associated with greater odds of online dating compared to heterosexual identification.

**Conclusion:**

Given the occurrence of online dating among early adolescents despite age restrictions, interventions might address age misrepresentation. Adolescent sexual health education may consider incorporating anticipatory guidance on online dating, especially for males and sexual minorities. Future research could further investigate online dating patterns from early to late adolescence and associated health effects.

## Introduction

The influence of real-time location-based dating apps has reshaped the landscape of socialization and dating. Approximately 25–39% of newly-formed couples have met through online dating apps [1, 2]. Previous studies on online dating have focused on adult or older adolescent samples [3, 4]. One study of U.S. adolescents and young adults (13–24 years) found that 19% of adolescents under age 18 reported using online dating sites, compared to 38% of young adults [5]; however, the mean age was 20 years and only 22% of the sample was under age 18. Another study of adolescents in urban environments (mean age 17 years) found that 10% of adolescents had initiated a romantic relationship online [6]. However, there is a paucity of studies examining the prevalence of online dating in early adolescence, which is an important developmental period characterized by more independence, the emergence of sexual feelings, and more concerns with body image and peer perception.

While the emergence of sexual feelings and initiation of dating may be a part of normal adolescent development, there are potential health risks associated with adolescent online dating. Early online dating debut (prior to age 18 years) in females was associated with higher anxiety and depression, as well as condomless sex [7]. Additionally, adolescents using online dating apps have reported experiencing insults, privacy violations, violence, and pressure for sex or sexual photos [8, 9]. Furthermore, studies suggest that online dating apps can be a platform to allow for the exchanging of sexually explicit or provocative content which is a risk factor for offline and online dating violence in adolescents [10, 11]. Childhood maltreatment, potentially through online dating, is a risk factor for sexual victimization in adulthood among young heterosexual women who use online dating, further elucidating the potential long-term effects of online dating at a young age [12].

Social epidemiology aims to examine how demographic (e.g., age, sex, race/ethnicity, sexual orientation) and socioeconomic (e.g., income, education) factors influence health and related factors in order to better understand health disparities [13–15]. Prior literature investigating socioeconomic and demographic correlates of adult online dating usage has documented that there is a higher prevalence of dating apps with user age ranges between 24 and 30 years of age, that about 60% of users are men, and that there may be a direct link between Tinder use and educational level [16]. One cross-sectional study of Canadian young adults found that Tinder use was associated with higher education (college/university vs. high school education), but was not significantly associated with gender, sexual orientation, or race/ethnicity [17]. However, demographic and socioeconomic factors associated with online dating in early adolescents remain unknown.

Given the rapidly evolving digital landscape and the increasing presence of younger demographics on social and dating applications, it is important to study early adolescents. This study aims to explore the social epidemiology of online dating in early adolescents using a demographically diverse national cohort of early adolescents. Findings may be important to inform digital literacy, health education, and guidance for early adolescents.

## Methods

### Study design

This analysis utilized cross-sectional data from Year 2 (2018–2020) of the Adolescent Brain Cognitive Development (ABCD) Study, a diverse, national cohort of adolescent health and development. The observational study recruited 11,875 at baseline (2016–2018, ages 9–10 years) from 21 study sites representing the nation’s major regions. Stratified, probability sampling of U.S. schools was informed by gender, race/ethnicity, socioeconomic status, and urbanicity to maximize representativeness of the baseline cohort with regards to the demographic and socioeconomic makeup of 9-10-year-old early adolescents in the U.S. For this analysis, we included 10,157 adolescent participants (mostly ages 11–12 years) with complete online dating and sociodemographic data at Year 2. Additional details regarding the ABCD Study’s recruitment process, procedures, participants, and measures have been described previously [18, 19]. Centralized institutional review board approval was obtained from the University of California, San Diego. Written informed consent and assent were obtained from a parent/guardian and the child, respectively, to participate in the ABCD study.

### Measures

Online dating was assessed through adolescent report of the following question, “Have you ever used a dating app?” (yes, no). Sociodemographic variables included parent report of biological sex (male, female), adolescent participant’s age, race/ethnicity (White, Latino/Hispanic, Black, Asian, Native American, Other), household income ($74,999 or less, $75,000 or greater, approximating the median household income in the US) [20], and highest parental education (high school education or less, college education or more). To assess sexual orientation, adolescents were asked, “Are you gay or bisexual?” (yes, maybe, no, don’t understand the question, decline to answer) [21]. Given small numbers, maybe, don’t understand the question, and decline to answer were combined into “other.”

### Statistical analysis

Multivariable logistic regression was used to estimate the associations between sociodemographic factors (e.g., sex, age, race/ethnicity, sexual orientation, household income, parental education) and online dating, adjusting for study site. Analyses were conducted in 2023 using Stata 18.0 (StataCorp, College Station, TX) and applied ABCD Study propensity weights to match key sociodemographic variables in the ABCD Study to early adolescents in the American Community Survey from the US Census [22]. This study followed the Strengthening the Reporting of Observational Studies in Epidemiology (STROBE) reporting guideline.

## Results

The sociodemographic and online dating characteristics of ABCD Study participants (mean age 12.04 years, 48.9% female, and 45.7% racial/ethnic minorities) are shown in Table 1. Overall, 0.4% (*n* = 38) reported ever using a dating app. Among early adolescents who reported ever using a dating app, 56.3% were male and 55.9% were White. Over a quarter (26.2%) of early adolescents who reported ever using a dating app were sexual minorities, whereas only 4.4% of early adolescents who had not used a dating app were sexual minorities.

**Table 1:** Sociodemographic characteristics among Adolescent Brain Cognitive Development (ABCD) Study participants 2018–2020 (*N* = 10,157)

Sociodemographic characteristics	Total	Online dating		*P*-Value
		No	Yes	
Age (years)	12.04	12.04	12.18	0.228
Sex (%)				0.569
Female	48.9%	48.9%	43.7%	
Male	51.1%	51.1%	56.3%	
Race/ethnicity (%)				0.005
White	54.3%	54.3%	55.9%	
Latino / Hispanic	19.8%	19.9%	9.3%	
Black	16.0%	15.9%	33.1%	
Asian, Native American, Other^a^	9.9%	10.0%	1.7%	
Sexual minority status				< 0.001
No	87.7%	87.7%	66.8%	
Yes	4.4%	4.4%	26.2%	
Other^c^	7.9%	7.9%	6.9%	
Household income				0.051
$75,000 or greater^b^	45.0%	45.1%	28.2%	
$74,999 or less	55.0%	54.9%	71.8%	
Parents’ highest education (%)				0.05
College education or more	81.6%	81.7%	68.2%	
High school education or less	18.40%	18.3%	31.8%	

Propensity weights from the Adolescent Brain Cognitive Development Study were applied based on the American Community Survey from the US Census

^a^ Asian, Native American, and Other race/ethnicity were combined due to small sample sizes

^b^ $75,000 approximated the median US household income during the study period

^c^ Other sexual orientation included: maybe gay or lesbian, don’t understand the question, decline to answer. These responses were combined due to small sample sizes

Adjusted associations with sociodemographic factors and online dating among ABCD Study participants are shown in Table 2. Male compared to female sex (AOR 2.72, 95% CI, 1.11–6.78) and sexual minority compared to non-sexual minority status (AOR 12.97, 95% CI, 4.32–38.96) were associated with higher odds of online dating, after adjusting for other sociodemographic factors.

**Table 2:** Sociodemographic associations with online dating among Adolescent Brain Cognitive Development (ABCD) Study participants 2018–2020 (*N* = 10,157)

Sociodemographic characteristics	Online dating	
	Adjusted odds ratio (95% CI)	*P*-value
Age (years)	1.32 (0.71, 2.48)	0.38
Sex (%)		
Female	reference	reference
Male	2.72 (1.11, 6.78)	0.03
Race/ethnicity (%)		
White	reference	reference
Latino / Hispanic	0.70 (0.19, 2.54)	0.59
Black	1.84 (0.68, 4.94)	0.23
Asian, Native American, Other^a^	0.16 (0.02, 1.38)	0.10
Sexual minority status		
No	reference	reference
Yes	12.97 (4.32, 38.96)	< 0.001
Other^c^	1.88 (0.52, 6.75)	0.33
Household income		
$75,000 or greater^b^	reference	reference
$74,999 or less	1.30 (0.47, 3.64)	0.60
Parents’ highest education (%)		
College education or more	reference	reference
High school education or less	1.41 (0.61, 3.28)	0.42

Propensity weights from the Adolescent Brain Cognitive Development Study were applied based on the American Community Survey from the US Census. Adjusted odds ratios represent the output from a logistic regression model including age, sex, race/ethnicity, sexual orientation, household income, parent education, and study site

^a^ Asian, Native American, and Other race/ethnicity were combined due to small sample sizes

^b^ $75,000 approximated the median US household income during the study period

^c^ Other sexual orientation included: maybe gay or lesbian, don’t understand the question, decline to answer. These responses were combined due to small sample sizes

## Discussion

In this large, diverse national sample of early adolescents (mostly 11–12 years old), we found that 0.4% reported ever using an online dating app. The prevalence estimate of online dating in early adolescents is significantly lower than the prevalence estimates previously reported in older adolescents (8–19%) [5, 6, 23] and young adults (38%) [5]. These trends are also in accordance with normal development, as sexual identity, intimacy, and one-on-one relationships are more characteristic of late adolescence and young adulthood than early adolescence [24]. Despite the low prevalence, the fact that any early adolescents have used online dating is notable since most online dating apps require that users be a minimum of 18 years old to join [25].

Early adolescent boys were nearly three times more likely to report using online dating compared to early adolescent girls. Studies in adults have shown that men are more active users of online dating than women, potentially due to their greater screen use and positive attitudes toward online dating [26, 27]. Early adolescent boys report 45 more minutes of screen use per day than early adolescent girls [28]. Among adolescents who used social media and had some relationship experience, boys were more likely than girls to report that social media made them feel more connected with their significant other (65% of boys versus 52% of girls) [23]. Furthermore, half of boys reported that social media made them feel more emotionally connected with their significant other, compared to only 37% of girls [23].

Sexual minority identity was associated with nearly thirteenfold higher odds of reporting online dating compared to heterosexual identification among early adolescents. Also, sexual minority early adolescents report nearly four more hours of daily recreational screen time than their heterosexual peers, across all modalities, including social media, texting, video chat, YouTube videos, and browsing the internet [29]. Sexual minority early adolescents may have fewer romantic partner options in their schools, where they may also face stigma and discrimination [26, 30]. Some dating apps are tailored towards sexual minority users, which may be valuable for identifying other sexual minority users, whereas the sexual orientation/identity of a potential partner may not be obvious in real life [26]. Dating apps often work with Global Positioning System (GPS) technology to connect users in close geographic proximity in real time, which may be particularly useful for minority users where there is a smaller local dating pool [31].

We did not find significant associations between race/ethnicity and online dating among early adolescents, similar to a prior study in young adults [17]. Household income and parent education, as proxies for socioeconomic status, were not significantly associated with online dating among early adolescents. Although some prior studies in adults have found that higher education level and higher income were associated with dating app usage [16, 17], this was referring to an adult’s personal socioeconomic status as opposed to that of their parents/households in the case of the early adolescent minors in this study. It should also be noted that dating app usage has been reported across socioeconomic backgrounds, including homeless youth in the US [32].

## Limitations

Limitations of this study include its cross-sectional nature and a limited number of early adolescents who endorsed online dating. Online dating was based on self-reports, which may be subject to recall, response, or social desirability bias. Sexual orientation was also based on adolescent self-report and the 11–12 year olds in this sample may not have a clear understanding of their sexual orientation or may not be out yet.

## Conclusions

To our knowledge, this is the first study to examine online dating among a national sample of 11-12-year-old early adolescents. We found that 0.4% reported ever using an online dating app. Given that sexual minority identification and male sex were associated with greater online dating, digital literacy and health education courses may consider anticipatory guidance focusing on these early adolescent populations. Future studies should explore online dating patterns (e.g., frequency, content) across early to late adolescence and determine downstream health effects (e.g., sexual, reproductive, and mental health).

## Data Availability

Written informed consent and assent were obtained from the parent/guardian and adolescent, respectively, to participate in the ABCD Study. Data used in the preparation of this article were obtained from the ABCD Study (https://abcdstudy.org), held in the NIMH Data Archive (NDA). Investigators can apply for data access through the NDA (https://nda.nih.gov/).

## Abbreviations

ABCD: Adolescent Brain and Cognitive Development
AOR: Adjusted odds ratio
GPS: Global Positioning Satellite
STROBE: Strengthening the Reporting of Observational Studies in Epidemiology
US: United States
